# Idiopathic Hypereosinophilic Syndrome Presenting As Eosinophilic Ascites and Pleural Effusion in an Elderly Male: A Rare Clinical Manifestation

**DOI:** 10.7759/cureus.90470

**Published:** 2025-08-19

**Authors:** Calvin Jose, Thomas V Pulickal, Issac Georgy, Ashley Alex, Avaroth Krishnadas

**Affiliations:** 1 Internal Medicine, Good Shepherd Hospital, Vythiri, IND; 2 Emergency Medicine, All India Institute of Medical Sciences, Bhubaneswar, Bhubaneswar, IND; 3 Internal Medicine, St. John's Medical College, Bangalore, IND; 4 Medicine, Yangzhou University, Yangzhou, CHN

**Keywords:** corticosteroids, eosinophilic ascites, eosinophilic gastroenteritis, hypereosinophilic syndrome, idiopathic hes, serosal eosinophilia

## Abstract

Hypereosinophilic syndrome (HES) is a rare disorder marked by sustained blood eosinophilia and associated tissue or organ damage in the absence of a secondary identifiable cause. Eosinophilic ascites and pleural effusion are extremely rare clinical presentations of idiopathic HES and often mimic malignancy, tuberculosis, or parasitic infections. We report a case of idiopathic HES in a 68-year-old male presenting with exudative eosinophilic ascites and pleural effusion. Extensive workup, including histopathology and bone marrow evaluation, confirmed the diagnosis. He responded favorably to corticosteroid therapy. This case highlights the importance of considering idiopathic HES in patients with eosinophilic effusions when common etiologies are excluded.

## Introduction

Hypereosinophilic syndrome (HES) encompasses a group of rare disorders characterized by persistent eosinophilia (>1,500/μL) for at least one month, with organ dysfunction attributable to eosinophilic infiltration, and without any identifiable underlying cause such as parasitic infections, allergic conditions, autoimmune diseases, or malignancy [1,2]. First formally described by Chusid et al. in 1975, HES was historically defined using a six-month time criterion, but this has since been modified to allow earlier diagnosis and intervention [3].

HES can be classified into several subtypes, including myeloproliferative, lymphocytic, familial, associated, and idiopathic forms, with idiopathic HES being the most prevalent in clinical practice, accounting for about 25-50% of cases [4]. Commonly affected organs include the skin, lungs, GI tract, heart, and nervous system, often with overlapping features [5].

Serosal involvement in HES, manifesting as eosinophilic pleural, pericardial, or peritoneal effusions, is exceedingly rare [6]. Such patients often undergo invasive procedures before a definitive diagnosis is reached.

We present a rare case of idiopathic HES in an elderly male, whose presenting features of ascites and pleural effusion were due to eosinophilic serositis. This case adds to the limited literature on such atypical presentations and emphasizes the importance of considering HES in unexplained eosinophilic effusions.

## Case presentation

A 68-year-old Indian male with a history of type 2 diabetes mellitus, hypertension, dyslipidemia, and hypothyroidism presented with a four-day history of progressive abdominal distension, bilateral pedal edema, and mild dyspnea. He denied fever, weight loss, jaundice, GI bleeding, recent travel, or use of new drugs.

Physical examination revealed shifting dullness and reduced breath sounds bilaterally at the lung bases. His vitals were stable (pulse rate 86 bpm, respiratory rate 16, blood pressure 130/80 mmHg, SpO₂ 98% on room air, afebrile). He had mild pedal edema, but no lymphadenopathy, hepatosplenomegaly, or rash was noted.

Laboratory investigations (Table 1) showed leukocytosis (WBC 12,270/µL), neutrophils 75%, eosinophils 9%, and an absolute eosinophil count (AEC) of 1200/µL. C-reactive protein was elevated at 103.8 mg/L. Stool analysis was also done (three separate stool samples taken for three separate days) with no evidence of intestinal parasitic infection. Autoimmune screening, including antinuclear antibody IIF and antineutrophil cytoplasmic antibodies testing, was negative. The echocardiogram revealed an ejection fraction of 62% and mild circumferential pericardial effusion with echogenic pericardial thickening.

**Table 1:** Laboratory investigations HB: hemoglobin, RDW-CV: red cell distribution width–coefficient of variation, MPV: mean platelet volume, WBC: white blood cell, RBC: red blood cell, PCV: packed cell volume, MCV: mean corpuscular volume, MCH: mean corpuscular hemoglobin, MCHC: mean corpuscular hemoglobin concentration, CRP: C-reactive protein, SGOT/AST: serum glutamic-oxaloacetic transaminase/aspartate aminotransferase, SGPT/ALT: serum glutamic-pyruvic transaminase/alanine aminotransferase, A/G ratio: albumin/globulin ratio, LDH: lactate dehydrogenase, SAAG: serum–ascites albumin gradient, PMN: polymorphonuclear leukocyte

Test name	Value	Unit	Reference range
HB	13.1	g/dL	13.2-16.6
RDW-CV	11.9	%	11.5-14.5
MPV	9	fL	7.4-10.4
Neutrophil	75	%	40-70
Lymphocyte	9	%	20-40
Eosinophil	9	%	1-6
Monocyte	7	%	2-10
Total WBC count	12270	Cell/Cumm	4000-11000
Absolute neutrophil count	9210	Cell/Cumm	1800-7700
Absolute lymphocyte count	920	Cell/Cumm	1000-4800
Absolute eosinophil count	1200	Cell/Cumm	20-500
Absolute monocyte count	920	Cell/Cumm	200-1000
Platelet count	5.27	Lakhs/cu mm	1.5-4.5 (150000-450000 /uL)
RBC count	4.55	millionCells/cumm	4.5-5.9
Haematocrit/PCV	37	%	38.3-48.6
MCV	83	fL	80-96
MCH	28	pg	27-33
MCHC	34	g/dL	33-36
CRP	103.8	mg/L	<10
Bilirubin total	0.8	mg/dL	0.3-1.2
Bilirubin direct	0.3	mg/dL	0.1-0.3
Bilirubin indirect	0.5	mg/dL	0.2-0.9
SGOT/AST	15	U/L	8-48
SGPT/ALT	16	U/L	7-55
Alkaline phosphatase	98	U/L	40-129
Total protein	6.9	g/dL	6.3-7.9
Albumin	3.6	g/dL	3.5-5.0
Globulin	3.3	g/dL	2.3-3.5
A/G ratio	1	Ratio	1.1-2.2
Urea	21	mg/dL	7-20
Creatinine	1.1	mg/dL	0.6-1.2
Uric acid	3.9	mg/dL	3.5-7.2
Sodium	120	mmol/L	135-145
Potassium	5.8	mmol/L	3.5-5.1
Ascitic fluid - albumin	2.5	g/dL	Varies; SAAG >1.1 for portal hypertension
Ascitic fluid - amylase	26	U/L	Similar to serum (<50 U/L typical)
Ascitic fluid - protein	4	g/dL	<2.5 (transudate), >2.5 (exudate)
Ascitic fluid - total count	1500	Cell/Cumm	<500 WBC/µL
Ascitic fluid - neutrophil	14	%	Varies; PMN <250/µL normal
Ascitic fluid - lymphocyte	17	%	Predominant in transudate
Ascitic fluid - eosinophil	69	%	<10% typical
Pleural fluid - albumin	2.1	g/dL	Gradient >1.2 g/dL (transudate)
Pleural fluid - LDH	238	U/L	<200 (transudate)
Pleural fluid - protein	4.3	g/dL	<3 (transudate), >3 (exudate)
Pleural fluid - neutrophil	12	%	Varies; low in transudate
Pleural fluid - lymphocyte	30	%	Predominant in many exudates
Pleural fluid - eosinophil	58	%	<10% typical
Pleural fluid - total count	625	Cell/Cumm	<1000 WBC/µL (transudate)

Ascitic fluid analysis revealed an exudative profile with a total leukocyte count of 1500 µL and 69% eosinophils; cytology was negative for malignancy. Pleural fluid showed 58% eosinophils. Cytology again ruled out malignancy.

**Figure 1:**
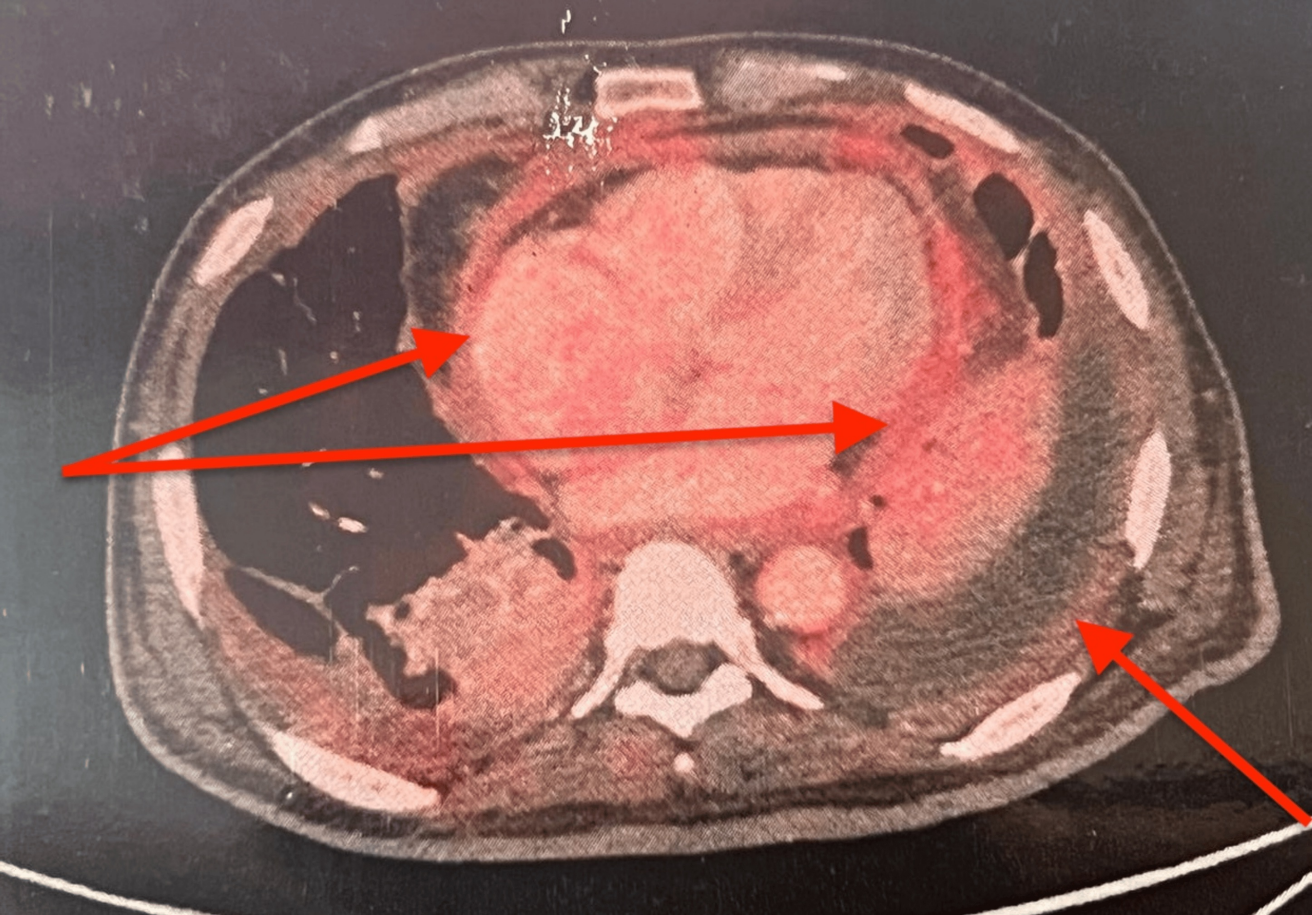
Low-grade FDG uptake with diffuse bilateral pleural thickening and effusion, pericardial thickening with effusion, and residual consolidation-collapse FDG: fluorodeoxyglucose

**Figure 2:**
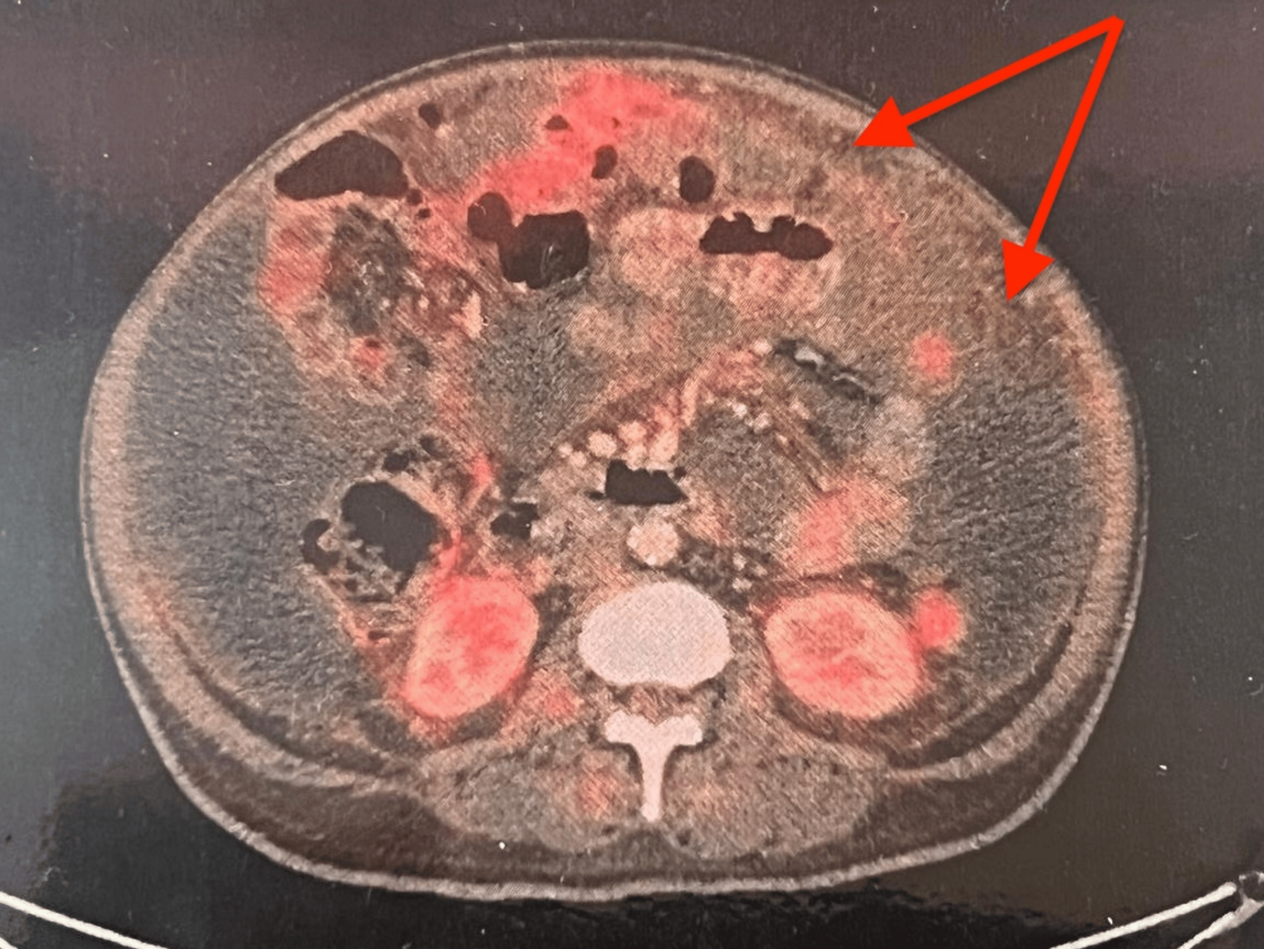
Low-grade FDG uptake with omental stranding and ascites FDG: fluorodeoxyglucose

Imaging with ultrasound showed coarse liver echotexture, a bulky pancreas, ascites, and bilateral pleural effusion. PET CT was done to rule out malignancy, which revealed low-grade avid fluorodeoxyglucose (FDG) uptake with ascites and omental stranding, thickened peritoneum, and bilateral pleural and pericardial thickening with effusion. His liver was normal in size and density, and the hepatic veins were normal (Figure 1, Figure 2). Previous collapse consolidation due to pneumonia was also visible.

Upper GI endoscopy and colonoscopy identified fundic and rectal polyps, which were benign (tubular adenoma with low-grade dysplasia), with no evidence of mucosal eosinophilic inflammation from multiple mucosal biopsies. Histopathological analysis of omental and peritoneal biopsies showed dense eosinophilic infiltration with no granulomas, malignancy, or parasitic elements. GI mucosal biopsies showed no eosinophilia. Bone marrow aspirate and biopsy revealed trilineage hematopoiesis with 20-25% eosinophils, hypercellular marrow, and no dysplasia or blasts, ruling out hematologic malignancy. Genetic studies were not done due to the patient's financial constraints.

Given the eosinophilic infiltration of serous membranes, absence of a secondary cause, and bone marrow findings, a diagnosis of idiopathic HES was made. Treatment was initiated with oral corticosteroids (prednisone) in a tapering dose starting from 60 mg/day, along with supportive therapy including diuretics, electrolytes, proton pump inhibitors, thyroxine, and glycemic control. The patient showed marked clinical improvement on steroid therapy over the coming weeks, with resolution of ascites and pleural effusion on an ultrasound test done a month later. Currently, he is on prednisone 7.5 mg/day.

## Discussion

We have presented a rare case of idiopathic HES manifesting as subserosal eosinophilic gastroenteritis (EGE) causing eosinophilic ascites with eosinophilic pleural and mild pericardial effusion, consistent with eosinophilic serositis. Although GI involvement is seen in about 25-35% of HES, true subserosal EGE with histologically confirmed eosinophilic ascites that is associated with multisystem involvement is very rare [4,6,7]. EGE is classified into three pathological subtypes according to the layer of predominant eosinophilic infiltration within the GI tract: mucosal EGE (most frequent, 25-100%), muscularis EGE (13-70%), and subserosal EGE (least common, around 12-40%) [8].

The subserosal type, defined by eosinophilic infiltration in the most external layer of the bowel wall and exudative eosinophilic ascites, is challenging to diagnose. Biopsy of upper GI endoscopy often only samples the mucosal layer, which makes it unsuitable for the diagnosis of subserosal EGE without mucosal infiltration [7]. In our case, even though the mucosal biopsies were unremarkable, the diagnosis was established by the peritoneal and omental biopsies that showed dense eosinophilic infiltrates, consistent with subserosal EGE.

The diagnosis was further corroborated by peripheral eosinophilia, as well as eosinophilic ascitic and pleural fluids. The peripheral eosinophilia was modest (AEC 1200/μL) at present, which can be seen in the early phase of HES or can fluctuate during the course [2]. Bone marrow studies demonstrated hypercellularity and increased eosinophils (20-25%), without dysplasia, fibrosis, or blasts. This ruled out myeloproliferative neoplasms, chronic eosinophilic leukemia, and other clonal disorders [9]. FIP1L1-PDGFRA fusion testing, cytogenetics, and flow cytometry were not performed in this case but are recommended to exclude the myeloproliferative variant of HES [10].

The possibility of lymphocytic HES was also considered but was unlikely. This subtype is associated with clonal proliferation of IL-5-secreting aberrant T-cells and frequently has skin lesions, lymphadenopathy, and a risk of progression to T-cell lymphoma [11,12]. No skin involvement, no lymphadenopathy, and no evidence of lymphocyte clonality on peripheral smear or bone marrow studies argued against this variant.

The pathogenesis of eosinophilic serositis, manifested as pleural and pericardial effusions, is a result of IL-5 and eotaxin-induced chemotaxis leading to capillary leak syndrome and tissue invasion [13]. According to the number of visceral serosal involvements (peritoneum, pleura, and pericardium), the present case is more consistent with idiopathic HES with subserosal EGE than with primary GI eosinophilic disease.

Corticosteroids represent the mainstay treatment of idiopathic HES and usually result in a prompt clinical response as a consequence of inhibition of eosinophil activation and survival. Steroid-sparing agents that can be considered in steroid-refractory patients or those in need of long-term treatment would include hydroxyurea, interferon-alpha, and imatinib, especially in PDGFRA-positive cases, and biologics such as mepolizumab (anti-IL-5) and benralizumab [14,15]. Our patient made a marked clinical recovery with corticosteroid therapy, which was further evidence in favor of the diagnosis of idiopathic HES. The pericardial effusion suggested an early multi-serosal involvement, highlighting the need for early therapeutic intervention to prevent irreversible end-organ damage due to eosinophilic myocarditis [4]. Greater recognition of subserosal EGE as a rare presentation of idiopathic HES is required, especially when eosinophilic ascites is seen with negative mucosal biopsies and systemic symptoms.

## Conclusions

HES remains a diagnostic challenge due to its rarity, heterogeneous clinical manifestations, and the need to exclude a wide array of secondary causes of eosinophilia. Recognition of pericardial effusion in our case indicated early multi-serosal involvement. It emphasized the urgency with which therapy should be initiated to prevent the development of irreversible end-organ damage due to eosinophilic myocarditis. This observation highlights the importance of clinical vigilance for early detection of serosal involvement, as this may precede more severe manifestations of HES. Such atypical presentations can often be overlooked, delaying diagnosis and treatment, which may lead to progressive tissue damage and poor patient outcomes. A high index of suspicion for HES in the presence of eosinophilia and serosal involvement is vital to ensure timely management and improve prognosis in these rare but potentially life-threatening cases.
